# In vivo validation of multimodality pore-network modeling to identify angio-permissive scaffold porosity

**DOI:** 10.1016/j.mtbio.2026.103377

**Published:** 2026-06-22

**Authors:** Andrea Tonelli, Francesco Iacoviello, Jaco Theron, Timothy Pennel, Peter Zilla

**Affiliations:** a Chris Barnard Division of Cardiothoracic Surgery, Department of Surgery, University of Cape Town, South Africa; b Cardiovascular Research Unit, Faculty of Health Sciences, University of Cape Town, South Africa; c Centre for Correlative Xray Microscopy, Electrochemical Innovation Lab, University College of London, United Kingdom

**Keywords:** Tissue-engineering, Angiogenesis, Vascularization, Micro-computed tomography, Porous media, Scaffold architecture, Structural connectivity

## Abstract

**Background:**

Successful vascular tissue regeneration in vascular and soft-tissue biomaterials is governed not by bulk volumetric porosity, but by the existence of continuous, ingrowth-permissive pathways traversing the full scaffold thickness, termed angio-permissivity. Conventional structural metrics often fail to capture these functional conduits, leading to unpredictable in vivo outcomes and a disconnect between scaffold design and biological integration.

**Methods:**

We developed a transmural space characterization workflow integrating micro-computed tomography, deep-learning–assisted super-resolution reconstruction and segmentation, and pore-network modeling. Three architecturally distinct electrospun scaffold groups were thresholded for continuous pathways (>10 μm) and analyzed for vascular ingrowth permissivity. Findings were validated in a subcutaneous rat model (7 and 21 days) to correlate architectural parameters with extracellular matrix remodeling and neovascularization.

**Results:**

Quantitative modeling identified a “porosity paradox,” where architectures with the highest total volumetric void space remained functionally isolated due to internal partitioning and sub-critical bottlenecks (<10 μm). Only scaffolds exhibiting a dense, continuous network of surface-to-surface growth tunnels supported robust transmural integration. *In vivo*, these continuous spatial configurations facilitated deep neovascularization and a rapid maturation “catch-up” to native glycosaminoglycan levels by day 21. In contrast, partitioned architectures restricted vessel recruitment and demonstrated impaired extracellular matrix preservation, regardless of high initial porosity.

**Conclusions:**

Transmural connectivity is a primary architectural determinant of tissue and vascular integration. This non-destructive, scalable framework enables the engineering-led design of tissue-engineered biomaterials by prioritizing the specific spatial and architectural requirements of the target physiological niche over stochastic volumetric metrics. Validation here uses a subcutaneous model that isolates architecture from cardiovascular hemodynamics — the next requirement for translation.

## Highlights

• PNM and super-resolution CT quantify transmural “angio-permissivity.”.
• High bulk porosity masks internal partitioning, creating a paradox of porosity.
• Continuous growth tunnels drive successful transmural vascular integration.
• Computational metrics correspond to in vivo neovascularization and matrix remodeling.
• A tunable engineering framework for predictive scaffold design and screening.

## Graphical abstract

Legend: The experimental workflow integrated advanced imaging, computational modeling, and in vivo validation to characterize scaffold performance. Initially, electrospun scaffolds were fabricated and imaged via micro-computed tomography to visualize 3D architecture. These data were processed through Dragonfly for fiber segmentation and analyzed using OpenPNM and a Python-based growth network algorithm to quantify pore and throat diameter distributions, thresholded to continuous network parameters. To assess biological integration, scaffolds were implanted subcutaneously in a murine model for up to 21 days. Following explantation, neo-vascularization was evaluated using CD31 immunohistochemistry—utilizing an HRP-polymer and DAB detection system—and analyzed via a digital slide scanner and automated segmentation algorithms to quantify vessel density and area.

**Figure:**
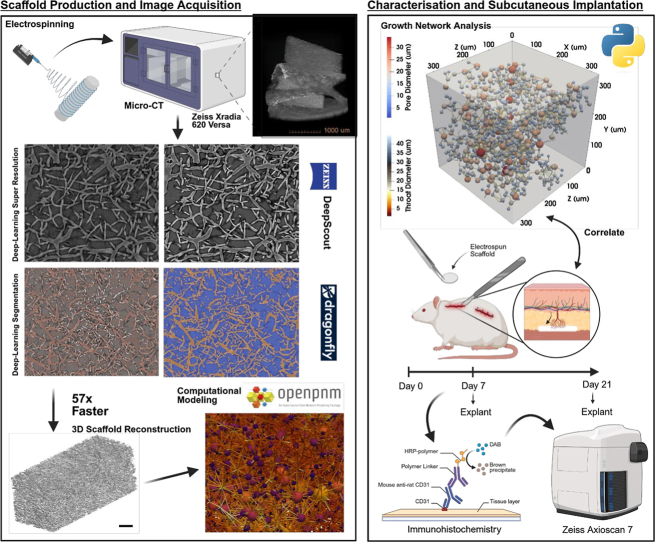

## 1 Introduction

The clinical viability of tissue-engineered cardiovascular and soft-tissue prostheses rests on their capacity for rapid and complete transmural integration [1]. For these biomimetic structures to function without structural failure or thrombosis, they must facilitate the transmural migration of host endothelial cells and the formation of progenitor-derived vascular regeneration and integration [[2], [3], [4]]. Despite decades of research into biomaterials, a fundamental challenge persists: our inability to accurately predict in vivo biological performance using conventional structural metrics [5,6]. Historically, the paradigm in scaffold design has prioritized bulk volumetric porosity and stochastic distributions of mean pore diameters as the primary benchmarks for cellular integration [7,8]. However these stochastic metrics are inherently deceptive; they provide an aggregate of isolated void spaces that lacks critical spatial context regarding the actual internal architecture. For example, Kohler et al. in 1992 showed how high porosity expanded polytetrafluoroethylene (ePTFE) vascular grafts most performant in animal models, failed to translate to man [9]. While bulk porosity and mean pore size quantify the total volume of void space, they do not resolve whether that space forms a functionally connected network capable of supporting vascular regeneration. Consequently, a reliance on volumetric averages frequently masks the **“porosity paradox”**—a phenomenon where architectures possessing high total void space remain functionally inaccessible to invading tissue due to internal partitioning and sub-critical geometric bottlenecks that preclude true **angio-permissivity**.

Vascular recruitment is not a function of total volume, but rather a result of the spatial continuity of specific architectural conduits [10,11]. A direct correlation between continuous transmural ingrowth spaces and vascularization has previously been demonstrated in porous scaffolds generated using well-defined porogens [[12], [13], [14]]. However, clinically applicable scaffolds must satisfy a broader set of constraints, including manufacturability, biocompatibility, scalability, and mechanical integrity. As a result, their architectures differ substantially from these experimental systems, which embody precisely defined, continuous void networks created by precision porogen techniques. Conventional scaffold materials, such as ePTFE, as well as more recent electrospun constructs, typically exhibit poorly defined transmural space continuity and are still largely characterized by the inadequate surrogate parameter of “effective porosity”, mean pore diameters and surface characterizations.

To bridge the gap between scaffold characterization and biological reality, we must move beyond stochastic descriptions of void space toward the quantification of **angio-permissivity**—the functional capacity of an architecture to permit unobstructed, through-thickness vascular integration. Leveraged extensively in hydrocarbon recovery and fuel cell engineering to simulate fluid flux through complex media, pore-network modeling (PNM) provides a robust framework for this task [15]. By treating the scaffold not as a static filter but as a dynamic system of pathways, PNM enables the high-throughput quantification of the continuous conduits through which cells, nutrients, and waste must migrate to sustain functional neo-tissue formation.

In this study we propose and test angio-permissivity as a candidate structural determinant of tissue integration, operationalized through a workflow that integrates whole-thickness micro-computed tomography with deep-learning–assisted super-resolution reconstruction and segmentation to identify and threshold surface-to-surface 'growth tunnels' based on a minimum viable inscribed diameter. We demonstrate that these continuous spatial configurations—rather than simple volumetric means—are the key determinants of transmural regeneration. By validating our computational metrics against a subcutaneous rat model—a well-established and ethically efficient platform that isolates scaffold architecture as the primary variable governing vascular ingrowth, independent of the hemodynamic forces of a cardiovascular implant site—we resolve the long-standing discordance between traditional structural analysis and functional integration, establishing a predictive, engineering-led characterization framework for soft-tissue vascular integration, with potential extension to other tissue-engineering applications contingent on further validation.

## 2 Methods

### 2.1 Materials

Materials: Pellethane® thermoplastic polyurethane (Lubrizol, 2363-80AE); DMF (Sigma-Aldrich, 319937-2.5L); THF (Supelco, 1.070-25.2500); Rhodamine B (Sigma-Aldrich, R6626-25G); hexane (Sigma-Aldrich, 34859).

### 2.2 Electrospinning

**Fig. 1:**
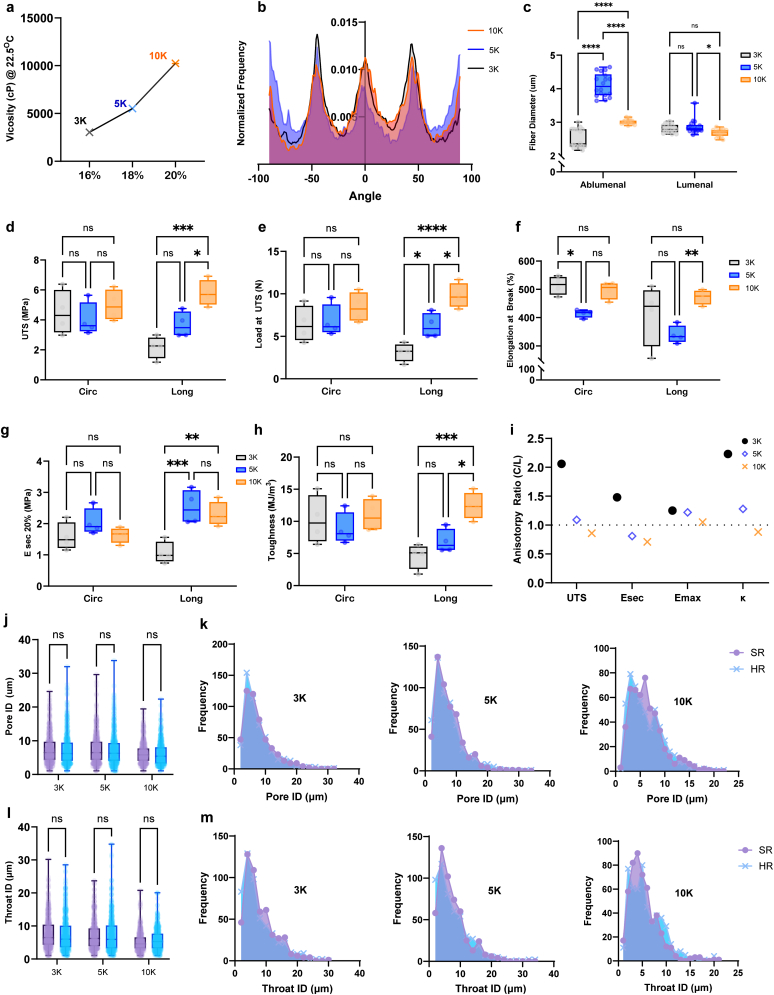
**Isolation of scaffold material properties as confounding variables.** Three electrospun groups, designated by mean solution viscosity (3K, 5K, 10K; corresponding to 16%, 18% and 20% w/w). **(a)** Rotational viscometry at 22.5°C defining the groups (3K = 3035, 5K = 5522, 10K = 10,240 cP; n = 3 per concentration; one-way ANOVA with Tukey's correction, all pairwise p < 0.0001). **(b)** Fiber orientation distributions (normalized frequency vs angle), closely overlapping and predominantly random across groups (n = 16 fields per group). **(c)** Abluminal and luminal fiber diameters (n = 18 fibers per group per surface; two-way ANOVA with Tukey's correction): abluminal diameters differed between all groups (3K: 2.52 ± 0.28; 5K: 4.11 ± 0.33; 10K: 3.01 ± 0.07 μm; all p < 0.0001), peaking in the 5K group, whereas luminal diameters were equivalent (3K: 2.81 ± 0.12; 5K: 2.86 ± 0.20; 10K: 2.69 ± 0.11 μm) apart from the 5K–10K pair (p = 0.032; 3K comparisons p ≥ 0.18). **(d**–**h)** Uniaxial tensile properties in circumferential (Circ) and longitudinal (Long) orientations (n = 4 per group per orientation; two-way ANOVA with Tukey's correction): (d) ultimate tensile strength, (e) load at UTS, (f) elongation at break, (g) 20% secant modulus (E_sec), (h) toughness (ĸ) **(i)** Anisotropy ratio (circumferential/longitudinal) for ultimate tensile strength (UTS), 20% secant modulus (E_sec), elongation at break (E_max) and toughness (κ). **(j**–**m)** Validation of deep-learning super-resolution (SR) against high-resolution (HR) micro-CT: pore (j) and throat (l) inscribed-diameter distributions were statistically indistinguishable between SR and HR within every group (ns), with frequency overlays in (k) and (m). Distributions were compared on random subsamples of up to 512 elements per group (GraphPad replicate maximum); complete extracted network sizes were — pores: SR 15,697/13,437/28,620 and HR 14,996/14,641/30,797; throats: SR 47,003/39,865/104,574 and HR 44,477/43,684/122,818 (3K/5K/10K). Statistical significance throughout: ns, p ≥ 0.05; ∗p < 0.05; ∗∗p < 0.01; ∗∗∗p < 0.001; ∗∗∗∗p < 0.0001.

Scaffolds were electrospun on a custom rig from Pellethane® solutions. Iterative variation of polymer concentrations (16%, 18% and 20% w/w) in DMF:THF (50:50) yielded three architecturally distinct scaffolds groups. To provide a single, unambiguous identifier independent of any downstream functional classification, groups were designated by their mean solution viscosity – 3K, 5K and 10K – corresponding to measured values of 3035, 5522 and 10 240 cP (22^°^C) at 16%, 18% and 20% w/w respectively (Fig. 1a). Solution viscosity was measured in triplicate on an Anton Paar ViscoQC 300 rotational viscometer with an RH6 spindle, with torque maintained within the instruments 60-67% window. The solutions were pumped (Chemyx Fusion 100) through PTFE tubing to an 18G blunt needle. Voltages were +18 kV (needle) and −4 kV (mandrel) (Gamma High Voltage Research, ES30P-30W/ES30N-30W) with a 25 cm working distance. Spinning was performed at 25°C and 45% relative humidity for 75 min on a custom-built rig. Additional rig parameters: mandrel diameter 26 mm; mandrel rotation speed 1000 rpm; oscillating traverse pattern; traverse speed 10 mm/s; chamber dimensions 80 cm × 60 cm x 60 cm (w x d x h). This viscosity ladder produced distinct fiber thickness, density and inter-fiber spacing.

### 2.3 Uniaxial Mechanical Testing

Uniaxial tensile testing was performed on dog-bone specimens die-cut from all three scaffold groups (3K, 5K and 10K; 16%, 18% and 20% w/w; n = 4 per group per orientation) in both circumferential and longitudinal orientations relative to the mandrel axis, in accordance with ASTM D882. Specimens had a gauge length of 21.82 mm and mid-segment width of 4.01 mm; mean thickness was measured prior to testing with a digital micrometer (Mitutoyo Corporation, Japan) and was 0.358 mm ± 0.090, 0.423 mm ± 0.087, and 0.422 mm ± 0.049, (16 replicates per group) for the 3K, 5K and 10K groups, respectively. Testing was conducted on a universal testing machine (Instron® 5544; 10 N load cell; rubber-lined self-aligning grips) at a crosshead speed of 50 mm/min following application of a 3% pre-strain to remove specimen slack. Ultimate tensile strength (UTS), elongation at break, Young's modulus (20% secant modulus, E_sec), and toughness (ĸ) (area under the stress-strain curve) were extracted from each stress–strain curve. Intergroup comparisons used two-way ANOVA with Tukey's post-hoc correction.

### 2.4 Imaging Techniques

To evaluate the accuracy of spatial reconstructions, micro-CT was compared against common imaging modalities, including scanning electron microscopy (SEM) and confocal laser scanning microscope (CLSM).

#### 2.4.1 SEM

SEM images were acquired on a JEOL JSM-IT100 InTouchScope™ (backscattered detector) at 500×, 15 kV, probe current 60.0, working distance 15.2 mm, and 70 Pa. A fixed acquisition recipe (contrast/brightness/stigmation/focus) was used throughout. Fiber diameters and orientation were quantified in Fiji (v1.51w) [1] using DiameterJ (v1-018) [2].

#### 2.4.2 Confocal

For CLSM, scaffolds were fluorescently labeled by adding Rhodamine B (0.15% w/w) to the polymer solutions before electrospinning. Z-stacks were acquired on a Zeiss LSM 880 AiryScan using a C Plan-Apochromat 63×/1.4 objective (561 nm excitation at 1.0%, pinhole 0.9 Airy units; emission collected to 632 nm; PMT gain 426; 1×1 binning). Image stacks (512×512 pixels; 224.92 × 224.92 μm field of view) were collected to the maximum detectable depth, segmented in Fiji using Otsu thresholding [1], and exported as TIFF stacks for analysis.

#### 2.4.3 Micro CT, Super-Resolution, Segmentation

Micro-CT was performed on a Zeiss Xradia 620 Versa. Low-resolution (LR) scans: 1601 projections/360° (adaptive motion compensation), voxel size 1.3852 μm, 50 kV/90 μA (4.51 W), 4× objective (geom. mag. 2.462×), binning 1, 2 s exposure. High-resolution (HR) scan: 2001 projections/360°, voxel size 0.5473 μm, 50 kV/90 μA (4.50 W), 20× objective (geom. mag. 2.462×), binning 2, 5 s exposure. Super-resolution (SR) reconstructions were generated from the LR datasets using DeepScout (Advanced Reconstruction Toolbox, Zeiss), trained on a single registered LR (1.3852 μm)/HR (0.5473 μm) voxel pair (upscaling 2.55×). Fidelity was validated against the HR ground truth by comparing SR- and HR-derived pore- and throat-size distributions within the same volume. All pore-network analyses were performed on the SR volumes, with HR reserved as the validation reference.

### 2.5 Densitometric Porosity Determination

Densitometric (DP) porosity was measured on 6 mm scaffold discs using an analytical balance with a density kit (Mettler-Toledo XS105). Samples were weighed in air and then in hexane (chosen for low swelling and scaffold penetration), and mean fiber volume was calculated from displaced hexane using the known liquid density.

Porosity was calculated as:

(1) $$P=1-\frac{V_{f}}{V_{T}}$$

where *P* is porosity, *V*_*f*_ is fiber volume, and *V*_*T*_ is total sample volume. Fiber volume was calculated as:

(2) $$V_{f}=\alpha \cdot \frac{m_{\text{air}\;}-m_{\text{hexane}\;}}{\rho_{\text{hexane}\;}-\rho_{\text{air}\;}}$$

where α is the buoyancy correction factor; *m*_*air*_ and *m*_*hexane*_ are masses in air and hexane; ρ_air_ = 0.001225 g/cm^3^ and ρ_hexane_ = 0.6837 g/cm^3^. Total volume was calculated from measured sample diameter and thickness (callipers):

(3) $$V_{T}=\pi {\left(d/2\right)}^{2}t$$

### 2.6 Growth Network Analysis

Reconstructed μCT image stacks were exported as sequential 8-bit TIFF files and processed using a custom Python pipeline (Tonelli, 2025; https://doi.org/10.5281/zenodo.20308262). Images were binarized using a fixed grayscale intensity threshold, and pore network extraction was performed using the SNOW2 algorithm implemented in PoreSpy v2.2.0 [16], which applies marker-based watershed segmentation to the distance transform of the binarized image. The resulting network was imported into OpenPNM v3.3.0^15^ and characterized using a spheres-and-cylinders geometry model. Threshold sensitivity analysis was performed by applying minimum inscribed diameter filters to pores and throats across a range of thresholds (5, 7.5, 10, 12.5 and 15 μm). Of these, 10 μm — corresponding to the lower bound of capillary caliber [17,18] — was designated the primary threshold for defining transmural permissivity. Following each thresholding step, only the largest percolating pore cluster was retained using graph-based connected component analysis (scipy v1.13.1), as isolated pore clusters that are not connected to the main network cannot support cell migration and are not functionally relevant to transmural scaffold growth. Pore and throat inscribed diameter distributions were exported for statistical analysis in GraphPad Prism (v10, GraphPad Software Inc.). For each retained network we quantified pore and throat inscribed diameters, pore coordination number (throats per pore), the constriction ratio (pore diameter ÷ mean adjoining throat diameter) and the throat aspect ratio (throat length ÷ throat diameter), and connectivity density (β-connectivity density): the number of independent, redundant loops in the percolating network — the first Betti number, β = (throats − pores + 1), where a loop-free tree has β = 0 — normalized to scaffold volume (per mm^3^). Derived from the network's Euler characteristic, it is the established topological measure of branching redundancy used in trabecular-bone and vascular microstructure analysis, and quantifies how many alternative routes a network provides beyond those required for simple connection; higher values denote a more richly interconnected, resilient network [19,20].

### 2.7 Subcutaneous Implant Study

#### 2.7.1 Experimental Design

Animal procedures were approved by the University of Cape Town Faculty of Health Sciences Animal Ethics Committee (024-016). Wistar rats (6–10 weeks; 200–250 g) were acclimatized ≥5 days. Analgesia (tramadol 5 mg/kg) was provided. Anaesthesia was induced with isoflurane (5% for 2 min) and maintained at 1.5% in 1.5 L/min oxygen. Under sterile conditions, six 1 cm dorsal subcutaneous incisions were made and bluntly dissected to create 1.5 cm pockets; scaffold discs were placed deep within each pocket; 6 scaffolds per group per timepoint, across 4 animals, per time point. Incisions were closed with 5/0 monofilament nylon using a buried intradermal mattress suture. Animals were euthanized at 7 or 21 days (5% isoflurane induction followed by intracardiac KCl). Explants were harvested with a 5 mm rim of surrounding tissue to preserve the interface. Group allocation and analysis were blinded via third-party coding, with random allocation of animals to groups.

#### 2.7.2 Histology, IHC and SEM

Explants were fixed in glutaraldehyde (SEM), zinc fixative (CD31 IHC; BD Biosciences), or 10% formaldehyde (histology). Samples were dehydrated, cleared (isooctane), paraffin embedded, and sectioned at 6 μm with scaffolds oriented perpendicular to the cutting plane. Stains included H&E, picrosirius red, modified Masson's trichrome (Verhoeff counterstain), and Safranin O. For IHC, sections were probed with mouse anti-rat CD31 (1:100; Biosynth, 10R-CD31GRT) and detected using a mouse-on-rat alkaline phosphatase polymer system (Biocare Medical, 623) with NBT substrate (Thermo Fisher Scientific, 34042). SEM samples were ethanol dehydrated and dried in hexamethyldisilane (Merck, 440191; 2 × 30 s). Slides were scanned on a Zeiss AxioScan 7 (40×) and analyzed in QuPath (v0.60).

### 2.8 Statistical Analysis

Normality and homoscedasticity were assessed using Shapiro–Wilk and Bartlett's tests. Parametric data are reported as mean ± 95% CI; non-parametric data as median (IQR, min–max). Group comparisons used two-tailed *t*-tests (parametric), Anova, or Wilcoxon rank-sum tests (non-parametric) as appropriate, with significance at *p* < 0.05. Data processing and visualization used GraphPad Prism (v11.0.0). For pore- and throat-level distribution analyses, where an extracted network contained more than 512 elements a random subsample of 512 per group was drawn to comply with the replicate limit of GraphPad Prism; all elements were retained where fewer than 512 were available. Network counts, densities and connectivity metrics were computed from the complete network.

## 3 Results

### 3.1 Isolation of Scaffold Properties

To confirm that polymer concentration did not introduce systematic differences in scaffold mechanical properties that could confound the architectural analysis, uniaxial tensile testing was performed across all three groups. Fiber orientation distributions overlapped closely between groups, indicating comparable, predominantly random alignment (Fig. 1b). Luminal (tissue-contacting) fiber diameters were closely matched across groups (3K: 2.81 ± 0.12; 5K: 2.86 ± 0.20; 10K: 2.69 ± 0.11 μm; n = 18 per group), with only the 5K–10K pair reaching significance (p = 0.032; all 3K comparisons p ≥ 0.18). Abluminal fiber diameter differed statistically between all groups (3K: 2.52 ± 0.28; 5K: 4.11 ± 0.33; 10K: 3.01 ± 0.07 μm; all p < 0.0001) and peaked in the 5K group rather than tracking polymer concentration (Fig. 1c). However, the largest pairwise difference (≤1.6 μm) is small relative to the length scale over which fiber diameter influences cell behavior and is confined to the non-tissue-contacting abluminal surface; notably, the best-integrating 5K group carried the thickest abluminal fibers. These marginal, surface-restricted differences did not translate into divergent bulk mechanical behavior, as detailed below.

Circumferential UTS was comparable across groups (3K: 4.49 ± 1.47 MPa; 5K: 4.00 ± 1.12 MPa; 10K: 4.98 ± 1.03 MPa; *p* = 0.40-0.79) and did not follow a monotonic relationship with polymer concentration. Circumferential E_sec was similarly indistinguishable between Groups 3K and 10K (1.58 ± 0.44 vs. 1.64 ± 0.24 MPa; *p* = 0.98) and Groups 5K and 10K shared near-identical wall thicknesses (0.423 vs. 0.422 mm). No consistent concentration-dependent trend was observed for elongation at break or material toughness in the circumferential direction. Statistically significant differences were confined to longitudinal UTS, which increased monotonically with concentration (3K: 2.18 ± 0.75, 5K: 3.67 ± 0.85, 10K: 5.79 ± 0.86 MPa; significant only for 3K vs 10K, p = 0.0003, and 5K vs 10K, p = 0.026; the 3K vs 5K difference was not significant, p = 0.13); however, the subcutaneous model employed here does not impose directional loading, limiting the physiological relevance of this finding. Collectively, these data confirm broad mechanical equivalence across the three scaffold configurations, supporting pore network architecture as the principal experimental variable (Fig. 1d–i).

### 3.2 Comparison of Imaging Techniques

**Fig. 2:**
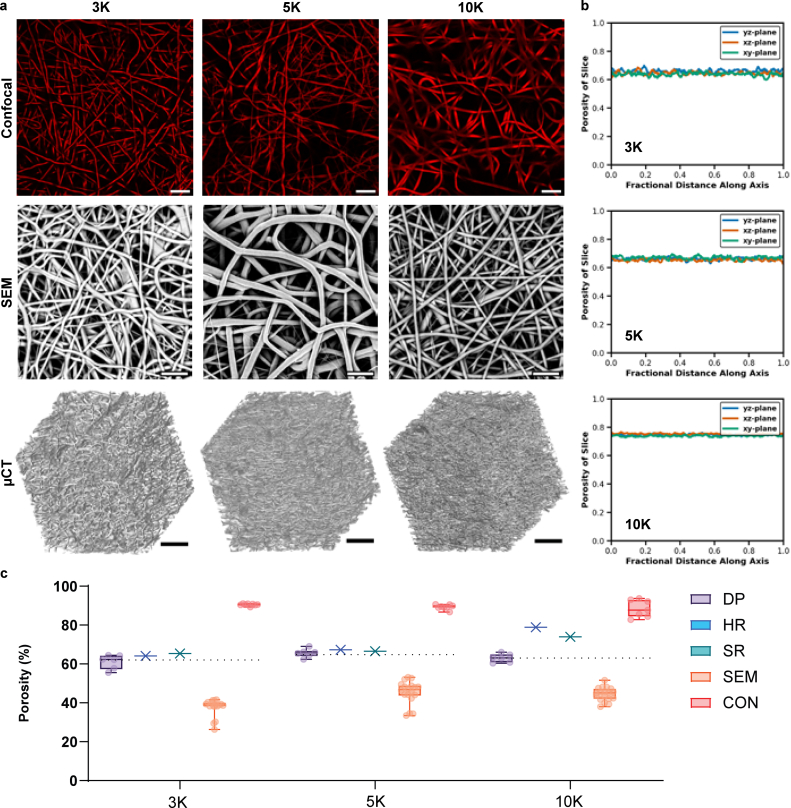
**Multimodal porosity characterization and the bulk-porosity paradox. (a)** Representative confocal laser scanning microscopy (CLSM; scale 25 μm), scanning electron microscopy (SEM; scale 25 μm) and micro-CT (scale 100 μm) of the 3K, 5K and 10K scaffolds; confocal data are shown for all three groups. **(b)** Transmural porosity profiles from high-resolution micro-CT (porosity of each slice vs fractional distance along the yz, xz and xy axes), demonstrating depth-independent structural homogeneity. **(c)** Porosity by modality and group: densitometric porosity (DP, n = 6 per group; dashed baseline), high-resolution micro-CT (HR, n = 1), super-resolution micro-CT (SR, n = 1), SEM (n = 17–18 per group) and confocal (CON, n = 7–8 per group); 95% confidence intervals shown for DP, SEM and CON. SR and HR agreed with DP (p ≥ 0.68 at 16% and 18% w/w), whereas SEM significantly underestimated and CLSM significantly overestimated porosity (both p < 0.0001 vs DP; two-way ANOVA with Tukey's correction). The 10K group carried the highest bulk porosity (SR 73.9%, HR 78.9%). Significance key as in Fig. 1.

**Fig. 3:**
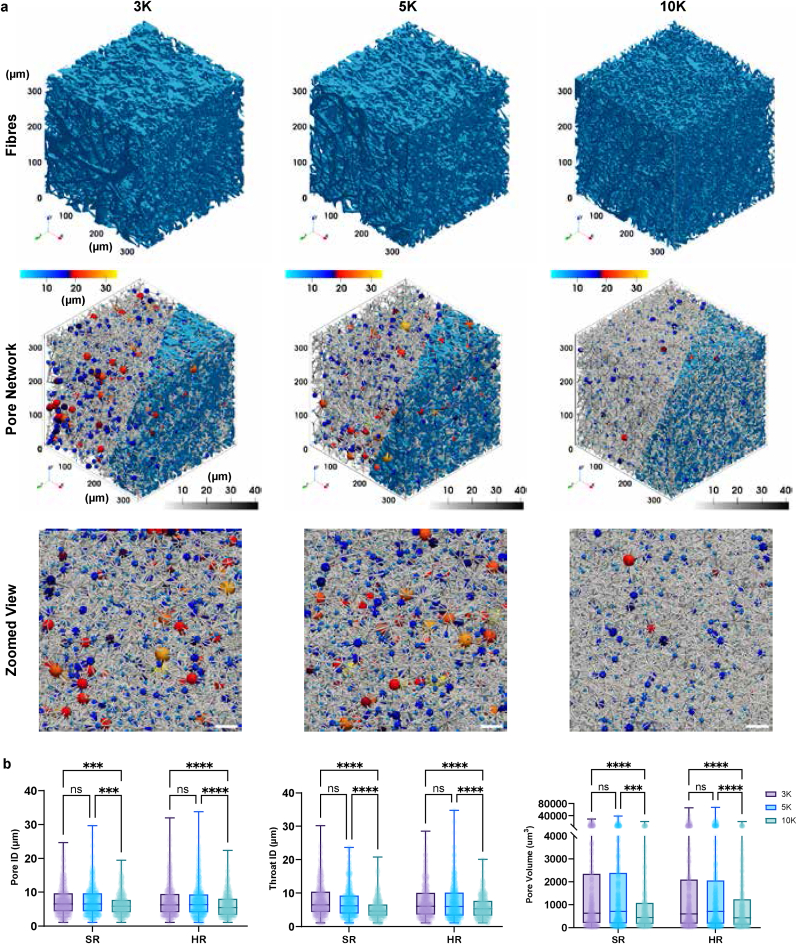
**Pore-network modeling of the transmural void space.** Pore-network models were extracted from super-resolution micro-CT volumes; each representative elementary volume (REV) was 600 [3] voxels (324 × 324 × 324 μm; ≈3.4 × 10^7^ μm^3^). **(a)** Three-dimensional renderings of the segmented Fiber phase (left), the overlaid pore network (center; pores = spheres, throats = cylinders, colored by inscribed diameter per the scalar bars) and a zoomed view (right; scale 25 μm); all axes in μm. **(b)** Pore inscribed diameter, throat inscribed diameter and pore volume, compared across groups within both SR and HR reconstructions; distributions were compared on random subsamples of up to 512 elements per group (GraphPad replicate maximum; complete network sizes as in Fig. 1; two-way ANOVA with Tukey's correction). The 10K group exhibited significantly smaller pores, throats and pore volumes than the 3K and 5K groups (p ≤ 0.001), which were mutually comparable (3K vs 5K, ns); SR and HR yielded concordant group rankings, confirming preservation of network topology. Significance key as in Fig. 1.

SR and HR micro-CT showed the closest agreement with DP porosity, whereas SEM significantly underestimated (p < 0.0001) and CLSM significantly overestimated (p < 0.0001) it, confirming that surface-biased and light-based imaging introduce systematic errors when quantifying dense transmural networks (Fig. 2c). Importantly, the deep-learning SR reconstruction was itself validated against the HR ground truth: SR- and HR-derived pore- and throat-size distributions were statistically indistinguishable (Fig. 1j–m), establishing that the network geometry analyzed in the following sections reflects the true architecture rather than a reconstruction artefact. Spatial analysis identified the partitioned 10K group as having the highest and most uniform through-thickness porosity (Fig. 2b); yet this volumetric advantage was not expressed as larger pores — the per-pore inscribed-diameter distributions did not track bulk porosity, the 10K pores being no larger than, and indeed somewhat smaller than, those of the lower-porosity groups (Fig. 3b). This dissociation between bulk porosity and pore caliber is the foundational observation of the 'porosity paradox' and mandates the connectivity-based PNM developed in the following sections to quantify effective, through-thickness regenerative space.

### 3.3 Pore-Network Modeling

To visualize the internal architecture of these regenerative spaces and assess their functional angio-permissivity, PNM was applied to the SR micro-CT datasets (Fig. 3). By translating the irregular geometry of the void space into a structured topological map of pores and throats, this approach revealed the stark structural contrasts that remain obscured by bulk measurements. Pore-network extraction revealed a marked divergence in topological complexity across three configurations. The 10K network reached the highest density, with 28,620 pores and 104,574 interconnecting throats—more than double the pore count and over 2.5-fold the throat count of the 5K configuration (13,437 pores; 39,865 throats). The 3K (15,697 pores; 47,003 throats) and 5K networks presented comparatively uniform, accessible geometries, whereas scalar mapping of the 10K network (Fig. 3a) revealed a highly intricate but congested pore space. Although the 10K group's mean pore and throat diameters were statistically the smallest of the three (Fig. 3b), the absolute differences were marginal — of the order of 1–2 μm against a shared ∼8–10 μm pore scale — a magnitude below any plausible threshold of biological consequence, irrespective of their statistical detectability. The operative distinction therefore lies not in mean pore size but in whether these pores form continuous, surface-to-surface pathways, as the following pore-network analysis resolves.

### 3.4 Growth Tunnel Analysis

**Fig. 4:**
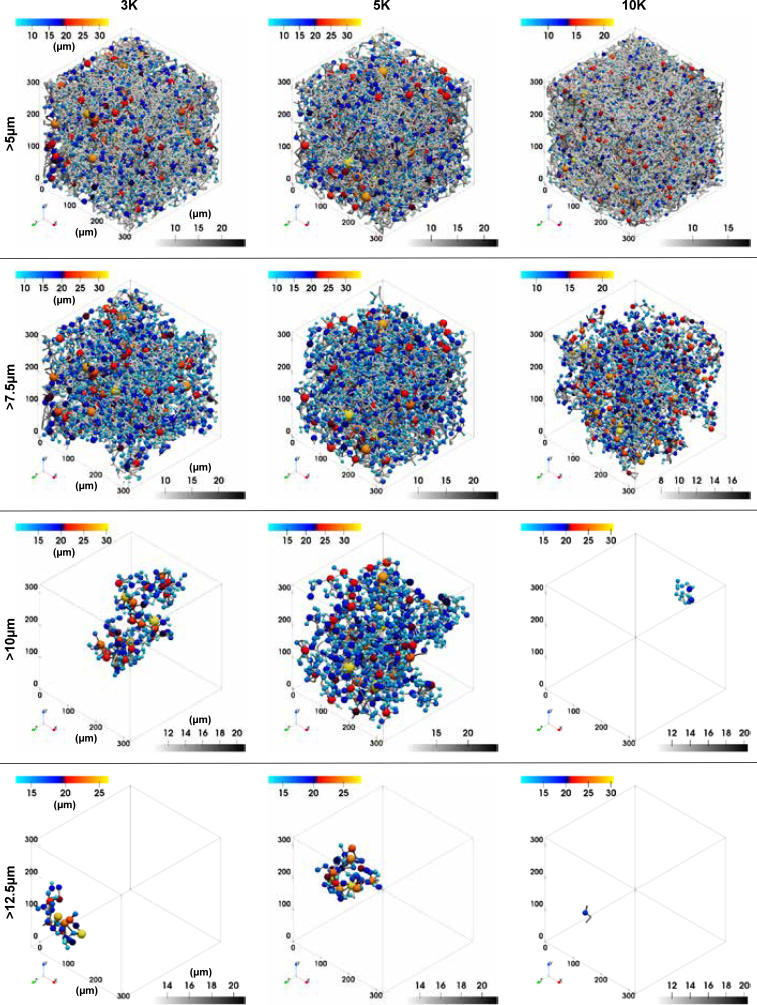
**Threshold sensitivity of the transmural growth-tunnel network.** Pore networks retained after applying minimum inscribed-diameter thresholds of >5, >7.5, >10 and > 12.5 μm to each group (3K, 5K, 10K), isolating only the continuous component. Spheres represent pores and cylinders represent throats, colored by inscribed diameter per the scalar bars; all axes and scale bars in μm. With increasing threshold the 3K and 10K networks fragment and collapse, whereas the 5K network retains a dense, continuous configuration up to and beyond the 10 μm capillary-relevant threshold — the qualitative basis for the sensitivity analysis quantified in Fig. 5.

**Fig. 5:**
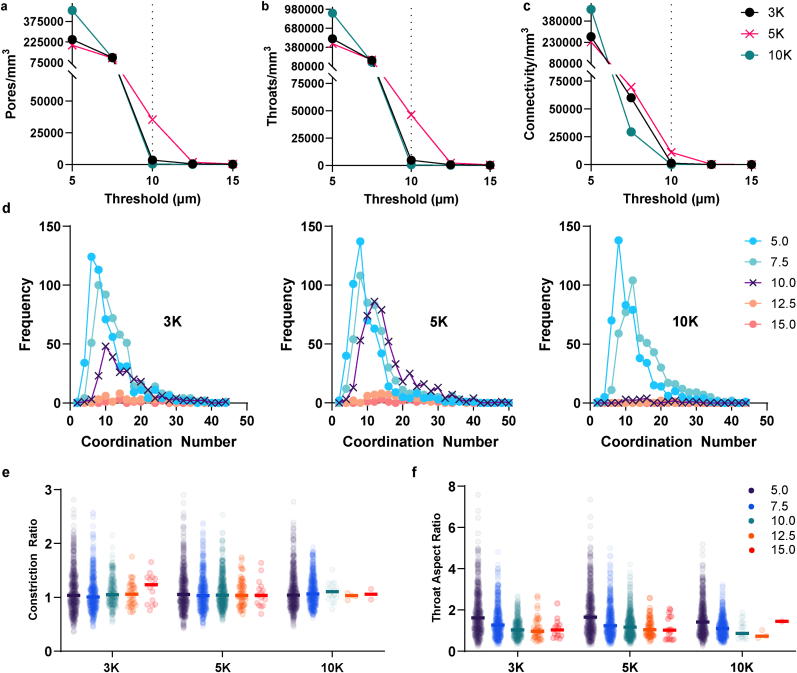
**Quantitative growth-tunnel analysis across thresholds.** Percolating pore networks were quantified across minimum inscribed-diameter thresholds of 5–15 μm (dotted line marks the 10 μm capillary threshold). **(a**–**c)** Density of the largest percolating cluster vs threshold, computed from the complete network: (a) pores/mm^3^, (b) throats/mm^3^, (c) connectivity/mm^3^ (β-connectivity density). At the 10 μm threshold the 5K network was an order of magnitude denser than either alternative (pore density 35,399 vs 3543 [3K] vs 500 [10K]/mm^3^; connectivity density 10,967 vs 1220 vs 118/mm^3^). **(d)** Frequency distributions of pore coordination number — the number of throats connected to each pore; higher values denote more branched, anastomosis-capable junctions — per group, colored by threshold (5.0–15.0 μm). **(e)** Constriction ratio (pore diameter/mean adjoining throat diameter) and **(f)** throat aspect ratio (throat length/throat diameter) per group at each threshold (each point = one pore or throat; bars = median). Distribution metrics (d–f) were compared on up to 512 retained elements per group per threshold (GraphPad replicate maximum); where fewer were retained — e.g. at 10 μm: 3K = 241 pores/323 throats; 5K = 1204/1576; 10K = 17/20 — all were used. Coordination-number comparisons used two-way ANOVA with Tukey's correction; significance key as in Fig. 1.

To resolve the functional connectivity obscured by bulk metrics, the pore-network models were filtered to isolate through-thickness “growth tunnels” - continuous, surface-to-surface pathways whose minimum inscribed diameter remained above a defined threshold — retaining only the largest percolating cluster at each step (Fig. 4, Fig. 5). At the 10 μm threshold the three architectures separated into quantitatively distinct classes. The 5K architecture was the sole angio-permissive (AP) network, retaining a dense, continuous, surface-to-surface configuration above 10 μm (35,399 pores/mm^3^; 46,336 throats/mm^3^; connectivity density 10,967/mm^3^) — an order of magnitude more connected than either alternative. The 3K architecture was constrictive (C): it carried a substantial sub-threshold network but failed to percolate above 10 μm, collapsing to a sparse connected component (3543 pores/mm^3^; 4748 throats/mm^3^; connectivity density 1220/mm^3^) — internal volume without transmural continuity. The 10K architecture was partitioned (P): despite the highest bulk porosity and the densest bulk throat population of all groups (916,582 throats/mm^3^ at 5 μm), it suffered the most severe collapse at 10 μm (500 pores/mm^3^; 588 throats/mm^3^ — an ∼1500-fold reduction in throat density; connectivity density 118/mm^3^), indicating that the overwhelming majority of its connections are sub-capillary (<10 μm) constrictions that isolate the internal pore space from the scaffold surfaces. Beyond connectivity density, the local topology of the conduits that remained patent was comparable across all three architectures and across thresholds: pore coordination numbers were high (medians 8–15 throats per pore), constriction ratios lay close to unity (median ≈1.05–1.10; throats only marginally narrower than their adjoining pores) and throat aspect ratios were low (median ≈1.0; short conduits) (Fig. 5d–f). To establish that this classification was not an artefact of the specific 10 μm cut-off, the analysis was repeated across thresholds from 5 to 15 μm (Fig. 4, Fig. 5). At the lowest threshold (5 μm) the high-porosity 10K group was in fact the densest network, consistent with its abundant small voids (454,337 pores/mm^3^ vs 242,604 [3K] and 202,633 [5K]); by 7.5 μm the three were comparable. This ranking then inverted entirely within the capillary-relevant window: at and above 10 μm the 5K network was the only one to retain a dense, percolating configuration, and it remained dominant at 12.5 μm while the 3K and 10K networks fragmented and collapsed (Fig. 4).

### 3.5 *In Vivo* Integration and Extracellular Matrix Remodeling

**Fig. 6:**
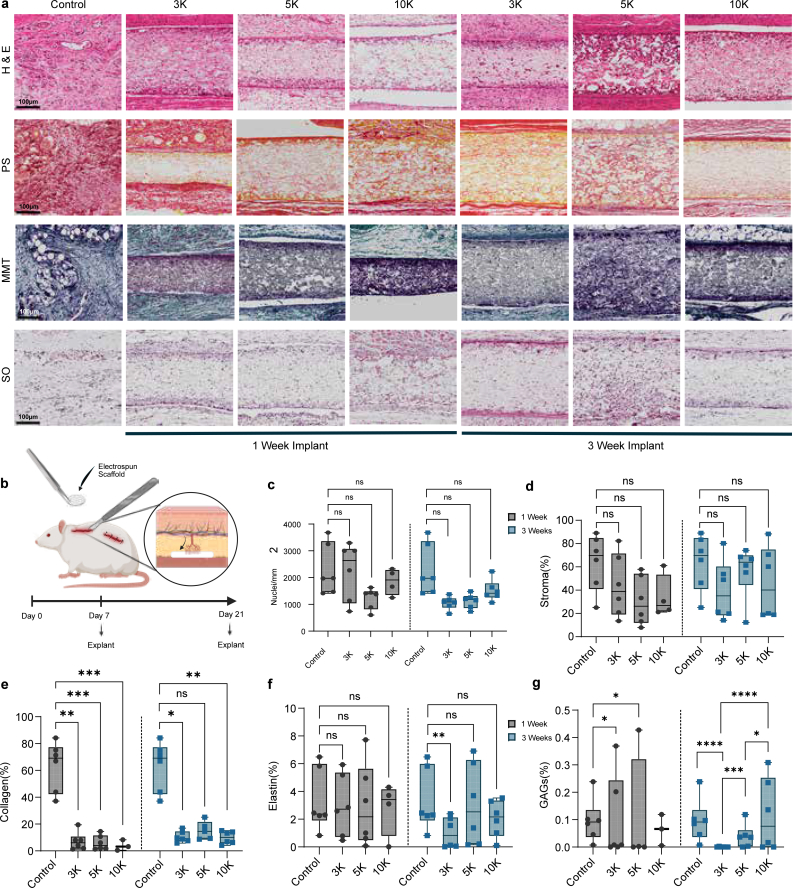
**Histological evaluation of subcutaneously implanted scaffolds. (a)** Representative sections of explanted 3K, 5K and 10K scaffolds and native-tissue controls at 1 and 3 weeks, stained with hematoxylin and eosin (H&E), picrosirius red (PS, collagen), modified Masson's trichrome (MMT, connective tissue) and Safranin O (SO, glycosaminoglycans); scale 100 μm. **(b)** Experimental timeline (subcutaneous implantation at day 0; explantation at days 7 and 21). **(c**–**g)** Quantification of (c) nuclei per mm^2^, (d) stroma %, (e) collagen %, (f) elastin % and (g) GAG %. n = 6 controls; 4 rats per timepoint, histology = 4-6 fields-of-view per scaffold per timepoint; occasional sections were excluded where tissue–scaffold separation precluded evaluation. Two-way ANOVA with Tukey's correction; data shown as box-and-whisker plots. Significance key as in Fig. 1. (For interpretation of the references to color in this figure legend, the reader is referred to the Web version of this article.)

Histological analysis confirmed that scaffold architecture shaped the temporal dynamics of matrix remodeling, while baseline cellular colonization was comparable across all constructs (Fig. 6). Cellular infiltration (nuclei/mm^2^) (Fig. 6c) and stromal fraction (Fig. 6d) showed no detectable difference between any scaffold group or the native control at either timepoint (all ns), indicating that the architectures recruited cells equally and that the divergent matrix and vascular outcomes below are not attributable to differential cell seeding. Collagen deposition was reduced in all three scaffold groups relative to native dermis at 7 days (3K p = 0.0018, 5K p = 0.0007, 10K p = 0.0008) (Fig. 6e), reflecting displacement of native tissue, with no difference between groups. By 21 days this deficit resolved selectively: the angio-permissive 5K group recovered to a level not statistically different from the native control (p = 0.19), whereas the constrictive 3K (p = 0.045) and partitioned 10K (p = 0.031) groups remained significantly below control. Elastin was preserved comparably across all groups at 7 days (all ns); by 21 days only the constrictive 3K group showed a significant reduction relative to control (p = 0.0091), the 5K and 10K groups retaining native-comparable levels (Fig. 6f).

Sulfated glycosaminoglycan (GAG) deposition followed group-specific timelines (Fig. 6g). At 7 days the partitioned 10K group mounted an early GAG response indistinguishable from native control (p = 0.89), while the 3K (p = 0.031) and 5K (p = 0.013) groups were significantly lower. By 21 days the angio-permissive 5K group had recovered to no detectable difference from control (p = 0.11) - a maturation 'catch-up' - but remained below the 10K group, which retained the highest GAG content of any group (5K vs 10K p = 0.032; 10K vs control ns, p = 0.88). The constrictive 3K group remained markedly GAG-deficient throughout (at 21 days: vs control p < 0.0001; vs 5K p = 0.0004; vs 10K p < 0.0001). The dissociation between the partitioned group's sustained, high GAG content and its poor vascular integration (below) is examined in the Discussion.

### 3.6 Vascular Tissue Regeneration

**Fig. 7:**
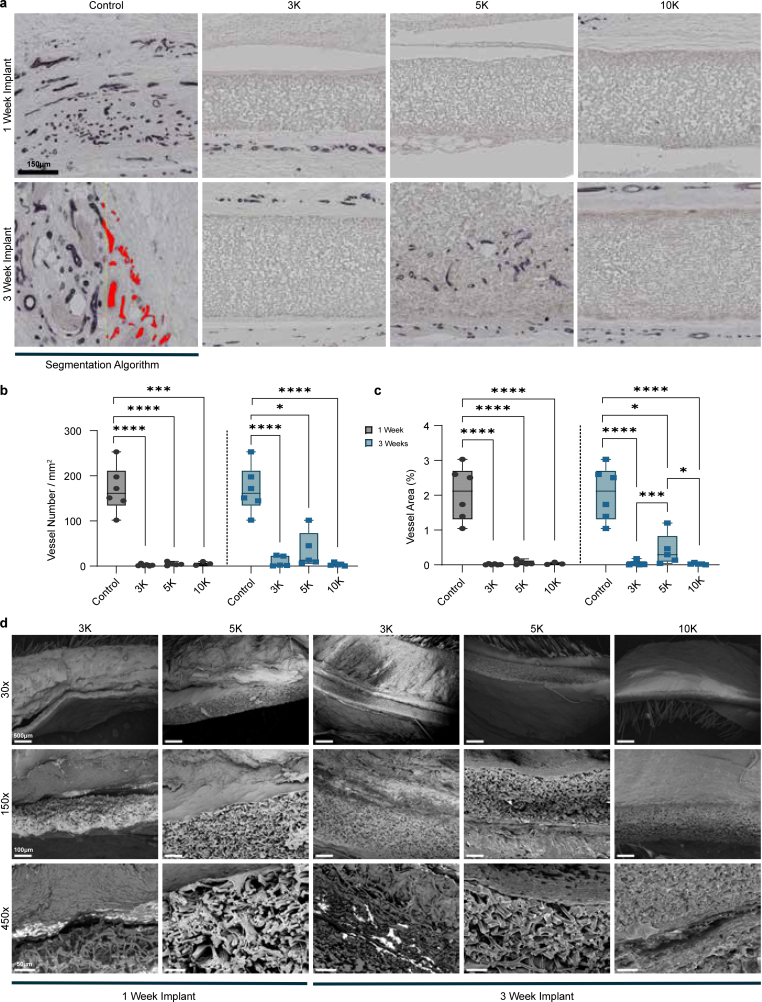
**Neovascularization and tissue–scaffold integration. (a)** CD31 immunohistochemistry of native controls and 3K/5K/10K explants at 1 and 3 weeks, with the automated vessel-segmentation overlay (red) shown for the control; scale 150 μm. **(b)** Vessel number per mm^2^ and **(c)** vessel area %, n = 6 controls; 4 rats per timepoint, histology = 4-6 fields-of-view per scaffold per timepoint; two-way ANOVA with Tukey's correction. **(d)** Cross-sectional SEM of the tissue–scaffold interface at 30× (scale 500 μm), 150× (100 μm) and 450× (50 μm). The 10K group is omitted from the 1-week SEM panel because the scaffolds separated completely from the surrounding tissue during explantation and processing. Significance key as in Fig. 1. (For interpretation of the references to color in this figure legend, the reader is referred to the Web version of this article.)

To test the physiological relevance of the growth-tunnel model, the predicted permissivity classes were correlated with vascular regeneration in vivo (Fig. 7). At 7 days vascular ingrowth was sparse and statistically indistinguishable across the three architectures, with vessel number (Fig. 7b) and vessel area (Fig. 7c) both far below native control in every group (all p < 0.0001 vs control; between-group comparisons ns) - consistent with an early phase in which architecture has not yet become rate-limiting. By 21 days the architectures diverged in the direction predicted by the network analysis. The angio-permissive 5K group was the best-vascularized scaffold and the only group to approach native control (vessel number p = 0.023; vessel area p = 0.027 vs control), carrying significantly greater vessel area than both the constrictive 3K (p = 0.0004) and partitioned 10K (p = 0.045) groups. The 3K and 10K groups remained strongly under-vascularized at 21 days, indistinguishable from one another (vessel area p = 0.64) and well below control (both p < 0.0001). Vessel number followed the same rank order but did not reach significance between groups (5K vs 3K p = 0.12; 5K vs 10K p = 0.16); the clearest architectural discriminator was therefore vessel area, and no scaffold matched the vascularity of native tissue at either timepoint.

High-resolution SEM corroborated this pattern (Fig. 7d): the angio-permissive 5K explants showed cellular infiltration throughout the scaffold core with intimate neo-tissue–fiber apposition, indicative of transmural integration, whereas the partitioned 10K architecture — despite its high bulk porosity and early provisional-matrix (GAG) deposition — confined vascular ingrowth to the superficial layers, and the constrictive 3K architecture remained sparsely infiltrated. This dissociation, in which the partitioned group's volumetric porosity and early matrix activity did not translate into deep vascularization, is the in vivo counterpart of the porosity paradox quantified in Fig. 5. The 1-week SEM panel does not include the 10K group, as the single explant allocated to SEM at this timepoint was lost during processing; quantitative histology and CD31 quantification for the 10K group at 1 week were unaffected and are reported above.

## 4 Discussion

This study resolves the discordance between traditional structural metrics and in vivo performance by introducing “effective porosity” - a metric quantified through continuous, surface-to-surface “growth tunnels.” By integrating deep-learning-enhanced micro-CT with PNM, we move beyond the qualitative description of fibers to the computational quantification of the interconnected pore space. We demonstrate that structural interconnectivity and geometric constraints, rather than simple volumetric or geometric means, are potentially the primary determinants of transmural tissue regeneration and vascularization. This workflow establishes a predictive framework for scaffold characterization, enabling the engineering-led design of biomaterials tailored to the specific migratory requirements of target physiological niches.

A central concern in attributing the divergent in vivo outcomes to pore-network topology is the potential co-variation of scaffold mechanical properties with polymer concentration — a variable known to regulate endothelial cell migration, macrophage polarization and angiogenesis through mechanotransduction [11,21], The tensile characterization presented here directly addresses this confound. Circumferential properties were statistically equivalent across all three groups (all p > 0.05) and did not increase monotonically with concentration; indeed, the angio-permissive 5K group recorded the lowest circumferential strength yet achieved the most favorable vascularization. The most instructive comparison is between the 5K and 10K groups, which shared near-identical wall thicknesses (0.423 vs. 0.422 mm) and statistically equivalent circumferential E_sec (2.05 ± 0.42 vs. 1.64 ± 0.24 MPa; p = 0.35) yet produced markedly different transmural integration; likewise the 3K and 10K groups — the two architectural extremes — exhibited near-equivalent circumferential stiffness (1.58 ± 0.44 vs. 1.64 ± 0.24 MPa; p = 0.98) while diverging substantially in neovascularization. Notably, fiber diameter did not scale monotonically with polymer concentration: the highest-concentration 10K group did not produce the thickest fibers but was significantly finer than the 5K group on both surfaces (Fig. 1c). This is consistent with an upper viscosity bound on stable electrospinning — fiber diameter increases with concentration only while a single, stable Taylor cone can be sustained, but once the viscous and surface-tension forces of the solution exceed what the applied potential can overcome, jet initiation destabilizes and the cone tends to split into multiple finer jets (and to branch during the whipping instability), redistributing the polymer mass into thinner fibers rather than a single thicker one [22,23].

These natural dissociations between mechanics and biological outcome are inconsistent with a substrate-stiffness-driven explanation. They are reinforced by scale: mechanotransductive regulation of angiogenesis and macrophage polarization is characterized at substrate stiffnesses in the pascal-to-kilopascal range [11,21], whereas the scaffolds studied here are far stiffer (circumferential E_sec ∼1.6–2.5 MPa), placing both the scaffolds and any sub-MPa inter-group difference well above the window over which cells discriminate mechanical cues. The same statistical-versus-biological distinction applies to fiber morphology: although abluminal fiber diameter differed between groups, the largest difference (≤1.6 μm) is small relative to the range over which fiber diameter modulates vascular cell behavior [22], while the tissue-contacting luminal surface was equivalent. Taken together — and consistent with the marginal, sub-2 μm differences in mean pore and throat caliber noted earlier — these data support transmural pore-network connectivity, rather than bulk mechanics or fiber morphology, as the operative variable governing the differential angiogenic response.

The 10 μm threshold used to define transmural growth tunnels is biologically conservative. It sits at the lower bound of capillary caliber (5–10 μm) [17,18] and above the nuclear-deformation limit below which cell migration physically arrests once a constriction narrows past the deformable size of the nucleus [24]. Because Pellethane is non-resorbable, cells cannot proteolytically widen these conduits; unlike in degradable matrices the geometric bottleneck is therefore a hard constraint on ingrowth, and a 10 μm path is the minimum that admits both a nascent capillary lumen and the migrating endothelial nucleus without reliance on matrix remodeling. The nearest experimental analog to this scale is the *in vitro* finding that endothelial cells still migrate through ∼9 μm pores during transmural endothelialization, albeit far more slowly than through 18–50 μm pores [25] consistent with the 5–10 μm caliber of a nascent capillary. In vivo, ePTFE grafts endothelialize across their wall only once the internodal distance exceeds ∼30–60 μm [26,27], but internodal distance is the node-to-node fibril span and is generally larger than the narrowest constriction a migrating cell must cross — the quantity our minimum-inscribed-diameter analysis measures directly — so a 10 μm cut-off represents a conservative, single-capillary geometric floor fully consistent with that literature. Additionally, this value is not a single arbitrary cut-off — the sensitivity analysis (Fig. 4, Fig. 5) identified the 5K network as the sole percolating, angio-permissive architecture throughout the capillary-relevant window (≥10 μm). One caveat applies: micro-CT was performed in the dry state, so in vivo hydration, swelling or tissue contraction could shift the effective inscribed diameters. The persistence of the 5K advantage across the 10–12.5 μm range provides a margin of confidence against modest dimensional change, but the magnitude of any wet-state change in Pellethane is addressed as a limitation below.

The 10 μm floor adopted here is specific to a non-resorbable polyurethane, in which the conduit geometry is effectively fixed over the implant period and the narrowest constriction acts as a time-invariant limit on ingrowth. This assumption does not transfer directly to materials whose architecture evolves in situ. In degradable scaffolds, hydrogels, and other dynamically remodeling systems, cell-mediated proteolysis, swelling and matrix turnover continuously alter effective pathway dimensions [28,29], so a constriction that is sub-critical at implantation may be widened [24] – or, through degradation-driven collapse, narrowed [30] – over time. For such materials the present static threshold is best interpreted as a time-zero lower-bound: a physiologically faithful criterion would need to be made time-dependent, coupling the inscribed-diameter analysis to the degradation and remodeling kinetics of the specific system (ideally with longitudinal hydrated-state imaging) so that angio-permissivity is assessed across the evolving geometry.

Against this criterion, the most significant finding of this work is the identification of a “porosity paradox” most evident in the *partitioned* group. Despite possessing the highest and most consistent bulk porosity measured by micro-CT (Fig. 2c) and transmural fractional porosity (Fig. 2b), this cohort failed to translate its volumetric advantage into superior vascular integration (Fig. 7). Average pore dimension therefore offered little predictive value for transmural success; the outcome-defining difference between the architectures was not the size of the individual pores but the continuity of the pathways connecting them. Literature suggests that while high porosity reduces the physical barrier to entry, a highly partitioned internal architecture creates functional “bottlenecks” that impede sustained migration, and thus spatial factors, like pore interconnection remain crucial for reliable tissue regeneration [31,32]. Our PNM analysis confirms this mechanical basis: the *partitioned architecture* exhibited the highest density of constrictive “throats” (<10 μm), effectively isolating the internal pore space from the scaffold surfaces. In contrast, the *angio-permissive* group was the only scaffold to maintain a dense network of through-thickness growth tunnels above the 10-μm threshold. Two further features sharpen this distinction. First, among the conduits that did remain patent above 10 μm, the local, proportional geometry — coordination number, constriction ratio and throat aspect ratio — was comparable across all three groups, so the discriminating variable was not the quality of the individual conduits but how many of them assembled into a percolating, surface-to-surface network. Second, the angio-permissive network was topologically redundant rather than merely continuous: its connectivity density was an order of magnitude higher, providing numerous alternative transmural routes that persist if individual throats are obstructed, whereas the partitioned architecture was reduced to a near-tree-like, effectively loop-free remnant. While PNM models offer an intricate reconstruction of the pore-phase architecture, they do not inherently identify the critical network bottlenecks or transmural “growth tunnels” essential for targeted vascularization. By isolating these continuous pathways, the workflow linked surface-to-surface connectivity above the capillary-scale threshold to the divergent in vivo outcomes examined below. This indicates that for transmural soft-tissue vascular integration — where vascularization via micro-vessel ingrowth is the goal — a continuous minimum-viable path is more critical than the total volume of available space or the statistical mean of pore diameters.

The GAG findings warrant cautious interpretation, because GAG abundance is not by itself a marker of productive regeneration. Early wound healing deposits a hydrated, GAG-rich provisional matrix dominated by hyaluronan and versican [33], and the sustained presence of this provisional matrix — rather than its orderly resolution into mature, vascularized tissue — can produce an equally high GAG signal. The best- and worst-integrating groups in this study separate along exactly this line. In the angio-permissive 5K group, GAG content was low at 7 days and recovered to a level indistinguishable from native control by 21 days, in step with the group's superior vessel area — the expected signature of a provisional matrix maturing on schedule. The partitioned 10K group, by contrast, sustained the highest GAG content of any group at both timepoints yet remained among the most poorly vascularized, with ingrowth confined to superficial layers. We interpret this dissociation as retention of a provisional, GAG-rich matrix within the group's poorly perfused internal pockets rather than as advanced remodeling; scaffold microarchitecture is a known determinant of the macrophage response [34], and the small, partitioned, poorly drained voids characteristic of this architecture plausibly favor a persistent provisional or foreign-body milieu over vascular resolution. This interpretation is inferential — the present study did not immunophenotype the infiltrate or stain specifically for hyaluronan and versican — and direct characterization of the macrophage and provisional-matrix response across these architectures is a clear priority for future work.

Pore size requirements in tissue engineering applications are diverse, dependent on the type of tissue, anatomical niche, local mechanical forces and preferred biological response [7]. For example, pore sizes as small as 1-4 μm provided sufficient space for wound-healing scaffolds [35], while larger diameters of 30-100 μm facilitate capillary regeneration [36,37], and in bone tissue engineering, pores as large as 100-300 μm are ideal for mature bone formation, while pores >500 μm have shown significantly less new bone growth [38]. Our findings align with the principle that interconnectivity independently influences vascularization [39], but they go further by demonstrating that mean pore geometries and bulk porosity alone may be a deceptive metrics. While current standards often focus on these averages, our results prove that the minimum inscribed diameter of a continuous path is a true limiting factor for transmural integration. The failure of both the constrictive and partitioned groups — the former additionally marked by significant elastin loss, both by an absence of continuous pathways despite high measured porosity —further underscores that architectures failing to provide these “tunnels” cannot sustain the mechanical and biological cues necessary for synchronized extracellular matrix synthesis and vascular regeneration.

A major hurdle in tissue engineering has been the lack of non-destructive, high-throughput, 3-dimensional characterization tools [40]. Traditional SEM is inherently limited by “surface bias,” where only the superficial fiber layer is visible, leading to inaccurate 2D extrapolations of 3D space. CLSM, while offering 3D insights, is often hampered by limited penetration depths (10–20 μm) and refractive index mismatches in scaffolds exceeding 300 μm in thickness, depending on density and optical clarity [41]. By utilizing micro-CT with SR reconstruction, we successfully overcame these depth and resolution constraints. This method enabled the non-destructive analysis of large Representative Elementary Volumes (REVs), in contrast to other, destructive 3D imaging techniques such as FIB-SEM, which require months of data acquisition to capture significantly smaller volumes at much higher spatial resolution—levels of detail that exceed the practical requirements for biomaterial applications [42].

Several limitations qualify these findings. Foremost, biological validation used a dorsal subcutaneous rat model, which isolates the architectural variable under spontaneous angiogenesis but lacks the pulsatile pressure, shear stress, blood contact and circulating endothelial progenitors of a true cardiovascular environment; on present evidence the angio-permissivity framework is therefore best regarded as a general predictor of soft-tissue vascular ingrowth, with applicability to high-pressure vascular grafts requiring confirmation in a vascular-interposition or aortic-patch model. Methodologically, micro-CT was acquired dry whereas the scaffold functions hydrated; Pellethane's low water uptake as a hydrolytically stable polyether polyurethane makes large dimensional change unlikely, where, *in vitro* electrospun conduits have shown a strongly hydrophobic surface (water contact angle: 115.3^O^ ± 0.2) and 30 day incubation in PBS lead to no discernible alteration in scaffold structure [43]. *In vivo*, electrospun Pellethane vascular conduits have been proven to remain both dimensionally and chemically stable in an infrarenal vascular graft model even out to 6 months [44].

The DP porosity reference captures only bulk void fraction, and orthogonal methods such as mercury intrusion porosimetry, permeability or tortuosity measurement would independently validate the network predictions. The SNOW sphere-and-cylinder abstraction was devised for isotropic granular media and only approximates the elongated voids of an electrospun mesh, so absolute pore and throat dimensions are model-consistent descriptors rather than exact values — though, applied identically across groups, the between-group differences and the threshold classification are unaffected. The SR model was trained on a single low-/high-resolution pair and validated against HR ground truth by pore- and throat-size agreement (Fig. 1j–m), but image-similarity metrics (SSIM, PSNR) were not reported and generalization to other architectures would require retraining; likewise the network metrics derive from one representative volume per group, with biological replication provided only for the in vivo outcomes. Finally, reported instances of 'no detectable difference' reflect a failure to reject the null at the present sample sizes, not formal statistical equivalence.

## 5 Outlook

Having established transmural connectivity as a candidate determinant of vascular integration, several priorities follow from this work. The framework now requires validation beyond the spontaneous-angiogenesis setting of the subcutaneous model - in flow-bearing vascular-interposition or patch models, under hydrated-state imaging, and across a wider range of fiber architectures and materials — together with direct phenotyping of the infiltrating cells to test the provisional-matrix interpretation advanced above. With those caveats, the connectivity-based approach is in principle portable to any regenerative target in which transmural vascular ingrowth is rate-limiting. A natural extension is the neuro-vascular-osteogenic axis of large bone defects, where establishment of a perfusing vasculature is a prerequisite for, and spatially coordinates, osteogenesis [45]; quantifying angio-permissivity could help distinguish bone scaffolds that merely possess pore volume from those that provide the continuous conduits vascular invasion requires. Because the analysis is non-destructive and geometry-based, it is not restricted to soft electrospun mats: it could be applied equally to the rigid, architecturally defined pore networks of 3D-printed scaffolds and of bioelectronic or drug-eluting constructs, where transmural vascular integration is similarly a precondition for function. Realizing this will require coupling the network metrics to cell- and tissue-specific thresholds and to the relevant mechanical and degradation behavior, but it points toward a characterization step that can screen architectures computationally before committing them to in vivo study.

## Code availability statement

The pore network extraction and analysis code used in this study is openly available at https://github.com/andtoni/growth-tunnel-analysis (Tonelli, 2025; https://doi.org/10.5281/zenodo.20308262), archived on Zenodo under MIT lisense, with detailed instructions. All pore network extraction was performed using PoreSpy v2.2.0^16^ and PNM was performed using OpenPNM v3.3.0,^15^ running on Python 3.12. Three-dimensional network visualization was performed using ParaView v5.13.3 (Kitware Inc.).

## Declaration of use of generative AI

During the preparation of this work the author(s) used *Gemini Pro* and *Claude Opus 4.8* to aid in improving the readability of the final, drafted manuscript. The author(s) reviewed and edited the content as needed and take(s) full responsibility for the content of the publication.

## Funding sources

This article was undertaken with generous funding from the Life Healthcare Group, the Skye Foundation, the 10.13039/501100009978Oppenheimer Memorial Trust and the Cecil Renaud Educational Trust in South Africa, and with an access grant from the National Facility for Xray Computed Tomography (NXCT) through 10.13039/501100000266Engineering and Physical Sciences Research Council (10.13039/501100000266EPSRC), United Kingdom, grant EP/T02593X/1.

Additionally, the work reported herein was also made possible through funding by the 10.13039/501100001322South African Medical Research Council through its 10.13039/501100015777Division of Research Capacity Development under the Bongani Mayosi National 10.13039/100018696Health Scholars Programme from funding received from the Public 10.13039/100018696Health Enhancement Fund/South African 10.13039/100009041National Department of Health. The content hereof is the sole responsibility of the authors and does not necessarily represent the official views of the SAMRC.

## CRediT authorship contribution statement

**Andrea Tonelli:** Conceptualization, Data curation, Formal analysis, Funding acquisition, Investigation, Methodology, Software, Validation, Visualization, Writing – original draft. **Francesco Iacoviello:** Data curation, Formal analysis, Investigation, Software, Visualization, Writing – review & editing. **Jaco Theron:** Conceptualization, Resources, Supervision, Writing – review & editing. **Timothy Pennel:** Project administration, Resources, Supervision, Writing – review & editing. **Peter Zilla:** Conceptualization, Methodology, Project administration, Resources, Supervision, Writing – review & editing.

## Declaration of competing interest

The authors declare that they have no known competing financial interests or personal relationships that could have appeared to influence the work reported in this paper.

## Data Availability

Data will be made available on request.
